# A retrospective study of the efficacy of sulbactam in the treatment of patients with extensively drug-resistant Acinetobacter baumannii infections

**DOI:** 10.1007/s15010-024-02307-9

**Published:** 2024-07-23

**Authors:** Jiaxin Yu, Baoshuang Zhang, Yang Yang, Wei Dou, Yuliu Li, Anji Yang, Xiao Ruan, Wei Zuo, Bo Zhang

**Affiliations:** 1 Department of pharmacy & State Key Laboratory of Complex Severe and Rare Diseases, Peking Union Medical College Hospital, No.1 Shuaifuyuan Wangfujing Dongcheng District, Beijing, 100730 China; 2 School of Pharmaceutical Science and Technology, Tianjin University, Tianjin, China; 3 School of Chinese Materia Medica, Tianjin University of Traditional Chinese Medicine, Tianjin, China

**Keywords:** Sulbactam, Single formulation, Carbapenem-resistant, High dose

## Abstract

**Purpose:**

Sulbactam (SBT) is one of the most significant treatments for patients with extensively drug-resistant Acinetobacter baumannii (XDR-AB). However, the efficacy and safety of SBT and its high dose regimen has not been well documented. This retrospective study aimed to assess the efficacy and safety of SBT-based treatment, particularly at high-dose (≥ 6 g/day), for XDR-AB infection.

**Method:**

A total of 52 XDR-AB infected patients treated with intravenous SBT at Peking Union Medical College Hospital were included. The primary outcome was 28-day all-cause mortality, while the secondary outcome was 14-day clinical response and the time of response. The formulation of SBT in our study is 0.5 g per vial.

**Results:**

Among the patients, the 28-day all-cause mortality rate was 36.5% (19/52), and the favorable 14-day clinical response rate was 59.6% (31/52). The 28-day mortality was independently associated coinfection with gram-positive bacteria (GPB) and a shorter duration of therapy. Patients with intracranial infection might have a longer survival time. A favorable 14-day clinical response was associated with the dose of SBT, and a longer treatment duration. However, the higher creatinine clearance (CrCl) associated with a worse clincal response. In addition, a higher SBT dosage was significantly correlated with a shorter time to clinical response. No adverse effects related were reported.

**Conclusion:**

The single-agent formulation of SBT emerges as a promising alternative for the treatment of XDR-AB infection, such as intracranial infection, particularly at high doses (≥ 6 g/day). Besides, longer duration of treatment correlates with higher survival rate and better favorable clinical response. Higher CrCl negatively correlates with favorable clinical response.

**Supplementary Information:**

The online version contains supplementary material available at 10.1007/s15010-024-02307-9.

## Introduction

Extensively drug-resistant Acinetobacter baumannii (XDR-AB) is a difficult-to-treat nosocomial infection and poses a significant challenge to public health in the 21th century [1]. Unlike other antibiotic-resistant bacteria, no treatments have been demonstrated to significantly reduce mortality or improve the outcome of patients with invasive XDR-AB infections [2, 3]. It was defined as non-susceptibility to all but two or fewer antimicrobial categories such as polymyxins and tigecycline [4, 5], that means XDR-AB is always carbapenem-resistant acinetobacter baumannii (CR-AB). The World Health Organization (WHO) and the Centers for Disease Control and Prevention have recognized CR-AB as a priority pathogen, based on the lack of effective treatment options and an urgent need for increased research [6, 7]. Recently, drug resistance rate of A. baumannii has consistently remained elevated, exhibiting a high resistance against various antibiotics. Moreover, conventional antibiotics such as polymyxin B (PMB) and tigecycline (TC) that are frequently used in the treatment of XDR-AB have demonstrated various side effects [8–10]. Consequently, there is an imperative need to evaluate of the efficacy of “legacy” antimicrobials [11].

Sulbactam (SBT), which is widely used in the treatment of A. baumannii, serves as a β-lactamase inhibitor capable of binding to Class A and C β-lactamases, then leads to the permanent inactivation of these enzymes through the formation of covalent acyl-enzyme species [12]. In addition to its role as a β-lactamase inhibitor, sulbactam possesses an intrinsic antibiotic activity against Acinetobacter spp through inactivating Penicillin-Binding Proteins (PBPs) 1 A, PBP1b, and PBP3 [13, 14]. Nowadays, sulbactam is available as either a single formulation or in combination such as ampicillin etc. [15], and the formulation of SBT in our study is 0.5 g per vial. For the management of Carbapenem-Resistant Acinetobacter baumannii (CRAB) infections, the Infectious Diseases Society of America (IDSA) recommends a high-dose regimen of sulbactam ranging from 6 to 9 g, since high-dose sulbactam enhance the likelihood of successfully reaching its PBP targets [16]. However, inadequate data are available to ascertain whether standard-dose sulbactam and high-dose sulbactam exhibit equivalent efficacy for the treatment of XDR-AB [16].

While some studies have evaluated the efficacy of sulbactam combinations against resistant bacteria, there is a paucity of research investigating the efficacy and safety of sulbactam as the single formulation for the treatment of XDR-AB. This study was conducted to evaluate the effectiveness and safety of the sulbactam single formulation-based treatment for the XDR-AB infection. Besides, we aim to determine whether high dose regimen of sulbactam correlates with a significantly positive clinical response in cases of XDR-AB infection.

## Materials and methods

### Patients and study setting

A retrospective observational cohort study was conducted at Peking Union Medical College Hospital, a tertiary teaching hospital in Beijing with a bed capacity of 2000, spanning the period from August 31, 2014, to March 01, 2024. The study aimed to investigate patients meeting specific inclusion criteria, which involves: (1) individuals aged 18 years or older (2) received SBT for 48 h or more; (3) identified through microbiological tests as having XDR-AB infection. Exclusion criteria were as follows: (1) SBT treatment lasting less than 48 h; (2) incomplete medical records during SBT treatment; (3) patients without XDR-AB infection. In cases where multiple SBT prescriptions were encountered for XDR-AB infection, only the initial episode lasting more than 48 h was considered for analysis. This study was approved by the Research Ethics Committee of the Peking union medical college hosptial in accordance with the Declaration of Helsinki and the enrollment of patients in the study was undertaken with a dual confirmation process to ensure accuracy and reliability of the data. In this study, the SBT formulation was 0.5 g per vial, and the manufacturer of SBT was North China Pharmaceutical Company.Ltd. SBT dosages were systematically adjusted in accordance with the renal function of each patient, adhering to the prescribed guidelines for SBT. The instruction of SBT single agent in China is the same as the SBT compound preparation in the Food and Drug Administration, with creatinine clearance less than 30 ml/min, the dosing interval is prolonged [17]. All enrolled patients were followed up for 28 days to record survival status.

### Data collection

All individuals receiving SBT were systematically documented in the ward pharmacy system of Peking Union Medical College Hospital, and patient enrolled was applied based on predefined inclusion and exclusion criteria. Demographic, length of hospitalization (including the duration of ICU stay), clinical details (diagnosis, pathogenic bacteria, clinical response) and other data were extracted from the electronic records of the Hospital Information System (HIS). In addition, comprehensive details regarding comorbidities, prior hospitalizations, surgical history, and antibiotic utilization within the 28 days preceding admission was extracted from admission records. To assess the severity of the underlying diseases, the Acute Physiology and Chronic Health Evaluation II (APACHE II) score was employed, with the highest score recorded within the initial 24 h of treatment.

### Definitions and patient outcomes

#### Definitions

The definition of a fourteen-day favorable clinical response in this study defined as clinical cure and clinical improvement. Clinical cure was the improvement of clinical signs and symptoms leading to the discontinuation of antimicrobial therapy. Clinical improvement was the partial alleviation of signs and symptoms coupled with the continuation or de-escalation of antibacterial therapy. Conversely, a fourteen-day unfavorable clinical response was the persistence of signs and symptoms, mortality, or the recurrence of infection. In the alternative analysis, an intermediate outcome, time to clinical response, was defined as the time between the onset of antibiotic therapy and the point at which clinical response had occurred [18]. Besides, the combination SBT therapy was defined as receiving a secondary agent for a duration exceeding 48 h.

### Outcomes

The primary outcome was 28-day all-cause mortality. The secondary outcome was 14-day clinical response and the time of response.

### Microbiology

Identification of micro-organisms was performed by matrix-assisted laser desorption/ionization time-of-flight mass spectrometry (MALDI-TOF/MS). The susceptibility of all strains to amoxicillin-clavulanate, amikacin, aztreonam, ampicillin-sulbactam, cephalosporins including cefoperazone-sulbactam and ceftazidime-avibactam carbapenems, fluoroquinolones, gentamicin, minocycline, piperacillin-tazobactam, trimethoprim-sulfamethoxazole, tigecycline, and polymyxin-B were systematically tested. Minimum inhibitory concentrations (MICs) were determined following the Clinical and Laboratory Standards Institute (CLSI) guidelines [19]. Extensively drug-resistant (XDR) status was defined as non-susceptibility to at least one agent in all but two or fewer antimicrobial categories [5], MIC > 8 mg/mL for imipenem and meropenem were defined as carbapenem resistance in Acinetobacter baumannii [19].

### Statistical analysis

Statistical analyses were conducted using SPSS (Version 26.0. Armonk, NY: IBM Corp). Normally distributed data are presented as mean ± SD and were analyzed through independent sample t-test. Non-normally distributed data were expressed as median values (quartiles) and analyzed by Mann–Whitney U-test. Categorical variables were represented as n (%) and differences were compared using chi-square test or two-tailed Fisher’s exact test. To identify risk factors for unfavorable clinical responses, multivariate regression analysis was performed in a reverse stepwise manner. Non-parametric tests were employed to compare differences between groups with non-normally distributed data, and Fisher’s Exact Test was utilized to compare rates between groups. A two-sided *P* value < 0.05 was considered to indicate statistical significance.

## Results

### Baseline data of 52 patients treated with SBT

**Fig. 1:**
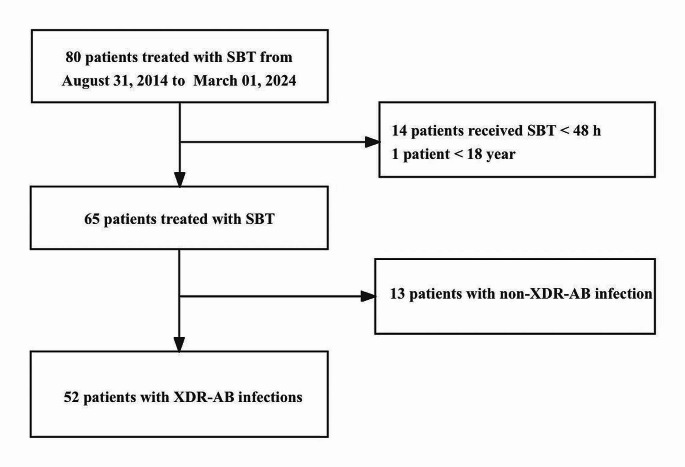
Flow chart of 52 patients treated with SBT

A total of 80 patients were initially evaluated, and ultimately, 52 patients were enrolled in this study (Fig. 1). The patients were classified into two groups (28-day survival group or 28-day died group) based on the primary outcome of 28-day all-cause mortality and baseline characteristics delineated in Table 1. There were 33 patients survived at 28 days while 19 died. The results demonstrated a higher incidence of mortality within the elderly patient, underlying chronic respiratory disease and patient who afflicted with bloodstream infections, possessing an APACHE II score ≥ 15, and those admitted to the intensive care unit during the treatment, *P* = 0.026, 0.037, 0.049, 0.029 and 0.030, respectively. Conversely, patients experiencing longer hospitalization and receiving extended duration of therapy exhibited high survival rates, *P* < 0.001. Patients who with pulmonary infection and pulmonary plus bloodstream infection, coinfected with GPB are less likely to survive, though there were no statistical difference, *P* = 0.053, 0.080 and 0.067. In addition, there is no adverse event in our study.

**Table 1:** Baseline Characteristics of Patients Survived and Died on 28 Day

Variables	Total (*n* = 52)	Survived (*n* = 33)	Died (*n* = 19)	*P*-value
Demographic parameters				
Age, yr (mean ± SD)	57.87 ± 18.41	53.61 ± 18.48	65.26 ± 16.20	0.026^*^
Male gender (n, %)	35 (67.3%)	24 (72.7%)	11 (57.9%)	0.272
BMI (mean ± SD)	23.81 ± 3.37	23.42 ± 3.46	24.51 ± 3.18	0.266
Concomitant diseases (n, %)				
Hepatic dysfunction	11 (21.2%)	8 (24.2%)	3 (15.8%)	0.714
Renal insufficiency	8 (15.4%)	5 (15.2%)	3 (15.8%)	1.000
Chronic respiratory disease (n, %)	23 (44.2%)	11 (33.3%)	12 (63.2%)	0.037^*^
Hypertension (n, %)	21 (40.4%)	12 (36.4%)	9 (47.4%)	0.436
Other cardiovascular disease	33 (63.5%)	19 (57.6%)	14 (73.7%)	0.245
Diabetes mellitus	17 (32.7%)	12 (36.4%)	5 (26.3%)	0.457
Immune compromise	13 (25.0%)	9 (27.3%)	4 (21.1%)	0.868
Infection site variables (n, %)				
Pulmonary infection (n, %)	44 (84.6%)	25 (75.8%)	19 (100.0%)	0.053
Plus bloodstream infection	11 (21.2%)	4 (12.1%)	7 (36.8%)	0.080
Plus urinary tract infection	1 (1.9%)	1 (3.0%)	0 (0.0%)	1.000
Bloodstream infection (n, %)	16 (30.8%)	7 (21.2%)	9 (47.4%)	0.049^*^
Intracranial infection (n, %)	9 (17.3%)	8 (24.2%)	1 (5.3%)	0.173
Microbiology characteristics (n, %)				
Coinfection variables				
Klebsiella pneumoniae	12 (23.1%)	8 (24.2%)	4 (21.1%)	1.000
Pseudomonas aeruginosa	7 (13.5%)	5 (15.2%)	2 (10.5%)	0.961
Concomitant with GPB infection	13 (25.0%)	5 (15.2%)	8 (42.1%)	0.067
Concomitant with fungi infection	27 (51.9%)	15 (45.5%)	12 (63.2%)	0.219
Treatment				
Length of hospitalization, (days, median, IQR)	32.0 (17.0 ~ 53.0)	51.0 (27.0 ~ 69.5)	18.0 (11.0 ~ 30.0)	< 0.001^*^
ICU duration (days, median, IQR)	16.0 (0.0 ~ 31.0)	12.0 (0.0 ~ 38.0)	16.0 (6.0 ~ 20.0)	0.939
Days of therapy (days, median, IQR)	6.0 (3.0 ~ 13.0)	11.0 (5.5 ~ 15.5)	3.0 (2.0 ~ 6.0)	< 0.001^*^
Hosptilized in ICU when SBT treated (n, %)	34 (65.4%)	18 (54.5%)	16 (84.2%)	0.030^*^
Time of respirator utilization	9.5 (2.25 ~ 19.5)	8.0 (1.0 ~ 21.0)	10.0 (6.0 ~ 19.0)	0.647
APACHE II ≥ 15 score (n, %)	28 (53.8%)	14 (42.4%)	14 (73.7%)	0.029^*^
CrCl (ml/min, median, IQR)	83.1(49.8 ~ 137.6)	83.9 (59.8 ~ 166.9)	66.3 (37.8 ~ 105.5)	0.141
Dual drug combination (n, %)	38 (73.1%)	24 (72.7%)	14 (73.7%)	0.940
With Tigecycline	19 (36.5%)	11 (33.3%)	8 (42.1%)	0.527
With polymyxin B	9 (17.3%)	5 (15.2%)	4 (21.1%)	0.872
With carbapenems	7 (13.5%)	6 (18.2%)	1 (5.3%)	0.372
With Minocycline	3 (5.8%)	3 (9.1%)	0 (0.0%)	0.462
triple drug combination (n, %)	14 (26.9%)	9 (27.3%)	5 (26.3%)	0.940
Dosage of sulbactam (g/day, n, %)	4.0(3.0 ~ 6.0)	4.0(3.0 ~ 6.0)	4.0(3.0 ~ 6.0)	1.000
≥ 6 g	18 (34.6%)	12 (36.4%)	6 (31.6%)	0.727
< 6 g	34 (65.4%)	21 (63.6%)	13 (68.4%)	0.727
Adverse event	0 (0.0%)	0 (0.0%)	0 (0.0%)	1.000

Note: ^*^P < 0.05, there was statistically significant difference.

Abbreviations: SD, standard deviation; BMI, body mass index; IQR, interquartile range; GPB, gram-positive bacterium; SBT, sulbactam; APACHE, acute physiology and chronic health evaluation; CrCl, creatinine clearance; ICU, Intensive Care Unit.

### Independent predictors of 28-day all-cause mortality and 14-day clinical response

Baseline characteristics of patients exhibiting favorable (*n* = 31) and unfavorable (*n* = 21) clinical response of 14 day were shown in Supplementary Table 1. The unfavorable response group demonstrated a higher body mass index (BMI), *P* = 0.013. In addition, the unfavorable response group comprised a higher proportion of patients with other cardiovascular diseases besides hypertension, lung infections, bloodstream infections, combined with GPB infections and hospitalized in ICU during the treatment compared to the favorable ones, *P* = 0.031, 0.032, 0.030, 0.014 and 0.002, respectively. Conversely, patients with longer treatment duration or long inpatient days were more likely to exhibit a favorite clinical response, *P* = 0.002 and 0.012, correspondingly.

**Fig. 2:**
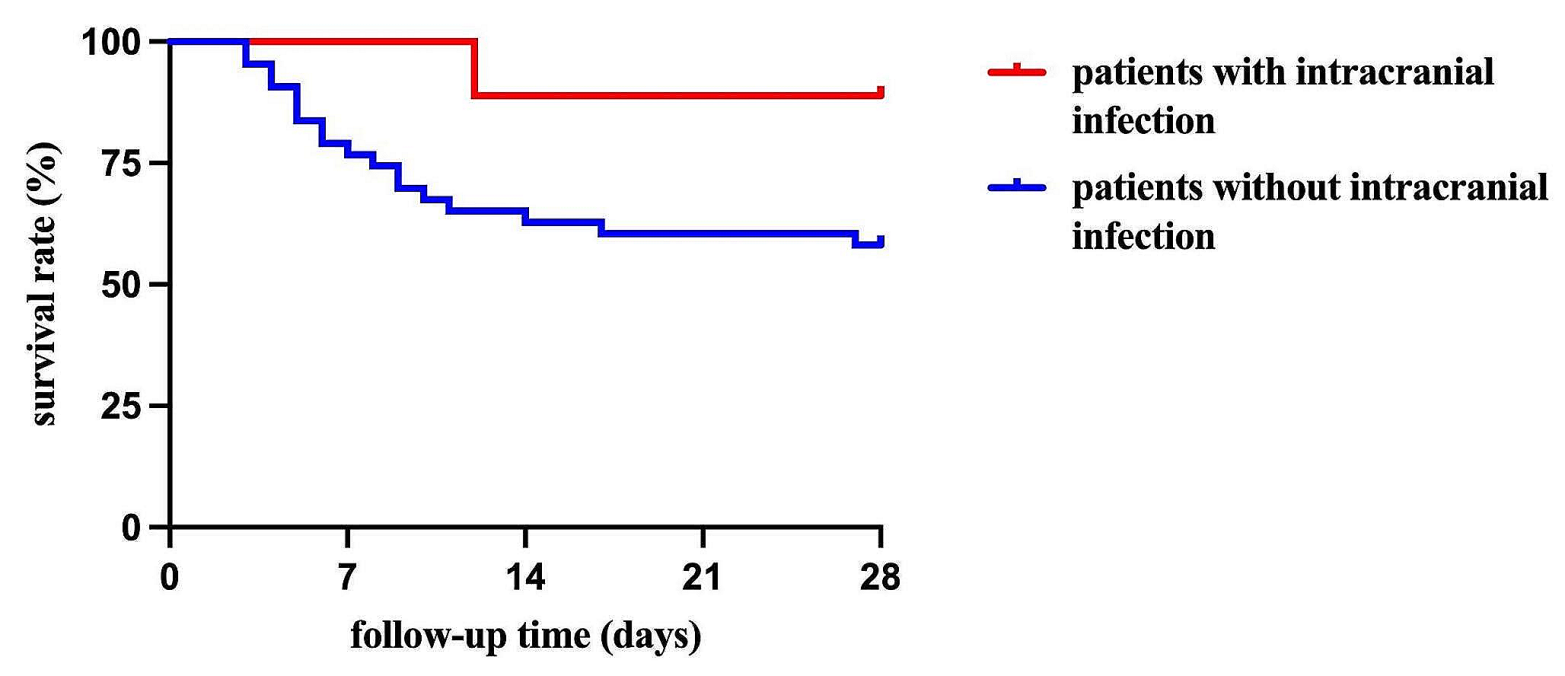
Survival curves of 52 patients with intracranial infection or not. Single trial with *n* = 9 (infected in intracranial), *n* = 43 (without intracranial infection); Log rank test was used to evaluate the difference. *P* = 0.09

Logistic regression analyses were conducted to examine variables affecting mortality by day 28, and clinical response by day 14. The multivariate analysis results were shown in Table 2, while the univariate analysis results can be found in Supplementary Table 2. In multivariate analysis, the 28-day mortality was independently associated with coinfection with GPB and a shorter duration of therapy. Clinical response was independently associated with duration of therapy and the dosage of SBT. Conversely, patients with high CrCl were identified as negative predictors of clinical response. The result in Table 2 shown that intracranial infection maybe a negative risk factor for 28-day death, OR value was 0.585. A Kaplan–Meier survival analysis was performed, using intracranial infection as a variable (Fig. 2). Intracranial infected patients had a longer survival time, *P* = 0.09, but without a significant statistical difference.

**Table 2:** Multivariate logistic regression analysis of variables associated with 28-day mortality and 14-day clinical response

	28-day mortality		14-day clinical response	
Covariate	*P*-value	OR (CI)	*P*-value	OR (CI)
Coinfected with GPB	0.019^*^	11.374 (1.501– 86.162)	-	-
bloodstream infection	0.234	2.595 (0.540–12.469)	0.119	0.274 (0.054–1.392)
intracranial infection	0.729	0.585 (0.028– 12.211)	-	-
Days of therapy	0.043^*^	0.847 (0.722–0.995)	0.013^*^	1.278 (1.054–1.550)
Hosptilized in ICU when SBT treated	0.294	3.356 (0.350– 32.180)	0.261	0.385 (0.073–2.031)
CrCl	-	-	0.038^*^	0.987 (0.976 − 0.999)
Dosage of SBT	-	-	0.021^*^	1.643 (1.079–2.502)

Note: ^*^P < 0.05, there was statistically significant difference

Abbreviations: GPB, gram-positive bacterium; ICU, Intensive Care Unit; SBT, sulbactam; CrCl, creatinine clearance

**Fig. 3:**
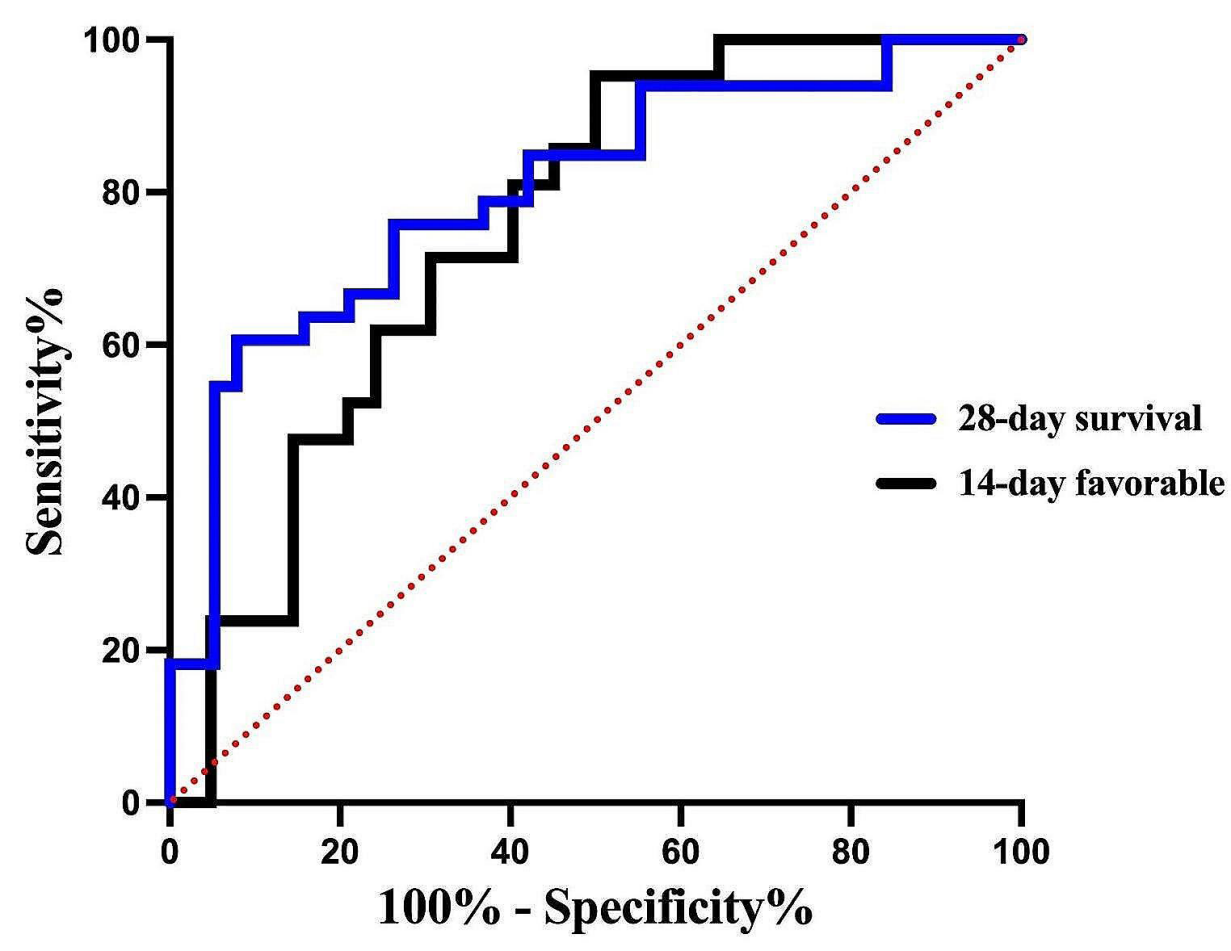
Receiver operating characteristic curves of the Days of therapy in SBT and 28-day survival and 14-day favorable clinical response for 52 patients

Besides, the 28-day survival and 14-day clinical response was significantly associated with the Days of therapy (DOT). To determine the cut-off values of the relationship between the DOT and survival as well as response, receiver operating characteristic curves of models were generated with AUCs of 0.803 (95% CI, 0.681–0.925) and 0.753 (95% CI, 0.624–0.883), *P* < 0.001 and *P* = 0.002, respectively, shown in Fig. 3. Youden’s index identified the optimal cut-off point as 8.5 days of survival by 28 day and 11.5 days of clinical response by 14 days. At these thresholds, the sensitivity was 60.6%, and the specificity was 89.5% in survival. For favorable clinical response, the sensitivity and specificity were 48.4%, and 95.2% respectfully.

### Higher dosage and normal dosage of SBT

**Fig. 4:**
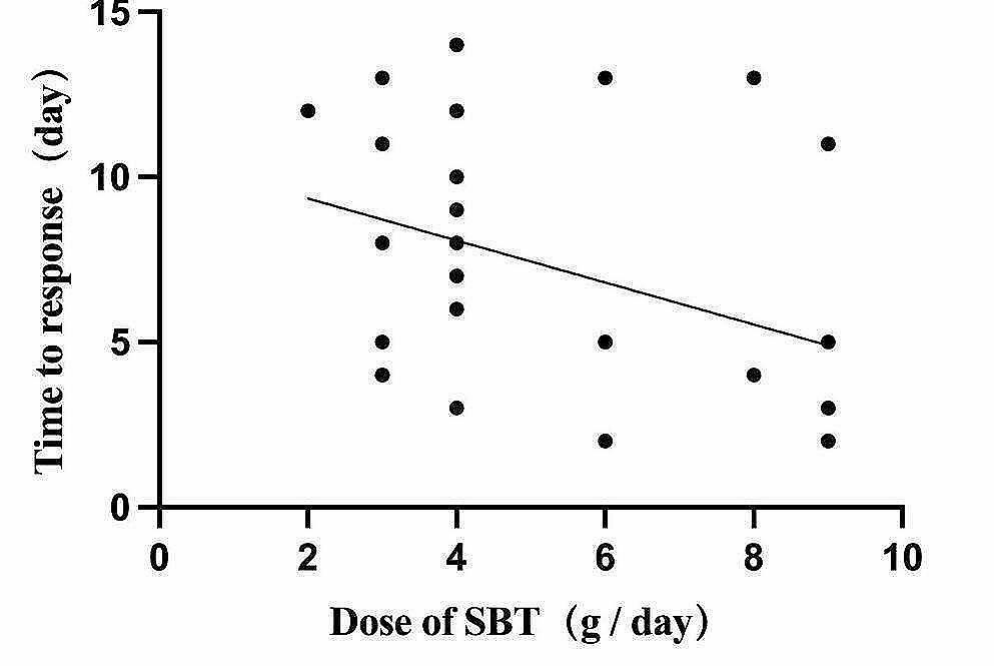
Relationship between the Dosage of SBT and time of response (*n* = 31)

The baseline characteristics of patients were detailed in Supplementary Table 3. There were 18 patients received higher doses (≥ 6 g/day), and 34 patients received normal doses (< 6 g/day). Our findings indicate that patients in the higher dosage group experienced a shorter time of response compared to the normal dosage group, *P* = 0.042. While no significant differences were observed in other baseline characteristics. To explore the correlation between dosage of SBT and time of response, Pearson correlation analysis was performed, 31 patients were included and 21 patients who exhibited an unfavorable response at 14 days were excluded. The results were shown in Fig. 4, it suggested that higher dosage was significantly correlated with shorter days of response, *P* = 0.03 and there was a weak negative correlation between the two variables, *r* = -0.381.

## Discussion

XDR-AB infection has been reported as a significantly causative agent of nosocomial infection, while the choice of therapeutic regimen is of great interest. For some patients, the treatment outcomes of the new antibiotics, such as PMB, tigecycline, etc. were not satisfactory. As a result, sulbactam, which is an old antimicrobial agent has gained more and more attention in recent years. However, it is noteworthy that only a limited number of countries, such as China offers the single-agent formulation of sulbactam. Furthermore, the majority of research on sulbactam has primarily focused on the combination dosage forms rather than the single-agent formulation. To our knowledge, this is the first clinical study focusing on sulbactam single agent in XDR-AB infection and evaluating the therapeutic efficacy of the high-dose sulbactam as a single agent formulation (≥ 6 g/day).

The all-cause mortality observe in our study was 36.5%. A study by Huang, S et al. on the treatment of pneumonia of Acinetobacter calcoaceticus-Acinetobacter baumannii complex with sulbactam (SBT) or ampicillin/sulbactam reported a similar mortality rate of 31.2% [15]. Besides, Niu, T et al. conducted a comparative analysis of CR-AB infected patients with bloodstream infection, treated with Cefoperazone/Sulbactam and Tigecycline. Their finding showed that the 28-day mortality rate of patients treated by Cefoperazone/Sulbactam was significantly lower than the those treated by tigecycline, 29.3% vs. 51.9%, *P* = 0.001 [20]. Similarly, Gu, S et al. reported a study of CR-AB infected patients treated with Cefoperazone/Sulbactam, demonstrating a significantly lower mortality rate in the Cefoperazone/Sulbactam group (25.4% vs. 53.4%, *P* = 0.005) [21]. In recent years, more and more research has been done on sulbactam-durlobactam [22, 23]. The result of study carried out by Karruli et al. showed that Sulbactam-Durlobactam can be an valuable option for patients with severe infections. Keith et al. had conducted a phase 3, non-inferiority clinical trial involving 181 patients randomly assigned to sulbactam-durlobactam or colistin due to carbapenem-resistant Acinetobacter baumannii-calcoaceticus complex (ABC) infection. This study demonstrated a lower 28-day all-cause mortality in patients receiving sulbactam-durlobactam compared to colistin group (19% vs. 32%).

Additionally, our study reported a clinical response rate of 59.6% (31 / 52) for sulbactam in the treatment of XDR-AB infected patients. In comparison, a study by G. Kalin et al. on patients with multidrug-resistant Acinetobacter baumannii ventilator-associated pneumonia indicated a 15-day clinical response of 43.2% to colistin/sulbactam treatment, but it’s worth noting that their study focused on patients with severe drug-resistant Acinetobacter baumannii ventilator-associated pneumonia [24]. In conclusion, sulbactam-based therapy may be a promising treatment to in improving both patient mortality and clinical response for XDR-AB infection. However, more research support is still needed for a comprehensive understanding of its effectiveness.

For the analysis of risk factors affecting 28-day mortality and 14-day unfavourable clinical response in patients with XDR-AB infections treated with sulbactam, our study firstly found that the duration of SBT therapy could affect the mortality and clinical response. This finding aligns with the study conducted by Huang, S et al., which suggested that a shorter duration of SBT-based therapy is a risk factor for clinical failure in patients [15]. Furthermore, receiver operating characteristic curves modeling indicated that the optimal cutoff for the duration of SBT treatment was 8.5 days for 28-day survival and 11.5 days for 14-day favorable clinical response, which was established for the first time and necessitates further investigation and validation in subsequent studies. Secondly, our study suggested that SBT-based therapy is likely suitable for patients who got intracranial infection, which demonstrated a longer survival duration by performing a Kaplan–Meier survival analysis, *P* = 0.09, without a statistical difference. The reason why maybe that only 9 patients with intracranial infection were enrolled in our study. In a study by Ye Y et al. involving 91 patients with carbapenem-resistant Gram-negative bacteria-related health care-associated ventriculitis and meningitis for 9 years, therapies of polymyxin and meropenem/sulbactam were considered optional choices [25]. Sun L et al. reported that ampicillin-sulbactam may be an effective therapeutic agent for meningitis caused by Acinetobacter baumannii resistant to imipenem and other β-lactam medication [26]. However, the majority of research has focused on SBT-based composite preparations, research of SBT single agent formulation was rare. Our study may provide a new therapeutic option for patients with intracranial infection.

Sulbactam which possesses intrinsic antibiotic activity, could saturate PBP1a/1b and PBP3 in A. baumannii isolates at high doses [12, 27]. The outcomes of our current study revealed that patient treated with a high dose of SBT (≥ 6 g/day) exhibit a shorter time of response consistent with the intrinsic properties of sulbactam as described above, *P* = 0.03 and *r* = -0.381. Moreover, the result of logistic regression analyses suggested that the CrCl of patients higher, the clinical response worse, which also manifested the importance of high dosage. Previous study indicated that the antibacterial activity of SBT depends on the time of its concentration remain above the minimum inhibitory concentration (MIC) [28]. A lower CrCl results in a higher likelihood of free concentration exceeding the percentage of time above MIC (%f T > MIC) of XDR-AB, therefore, potentially influences the clinical response to our antibiotics. This correlation may explain why a higher CrCl is associated with a less favorable clinical response in this study. Additionally, Infectious Diseases Society of America (IDSA) 2023 Guidance suggested high-dose ampicillin-sulbactam (sulbactam component 6–9 g/day) combine with at least one additional for treating CR-AB infections [16]. Another study indicated that in patients with XDR-AB infected pneumonia, treatment with colistin combined with sulbactam at a dosage of 12 g/day was not superior to 9 g/day [29], aligning with IDSA guidelines. Besides, a meta-analysis demonstrated that high-dose sulbactam (≥ 6 g/day) combining with other antibacterial medications such as colistin might might be a potential therapy for MDR-AB or XDR-AB infections [30]. But limited evidence on the safety of high-dose sulbactam therapy, existing findings indicate that high-dose ampicillin-sulbactam (sulbactam component 12 g/day) has caused no relevant adverse effects [31], while, another analysis suggested that patients treated with a daily dose of sulbactam/ampicillin of ≥ 9 g (sulbactam component 6 g/day) were more likely to experience an increase in alanine aminotransferase grade [32]. There is still a debate about safety of SBT. Moreover, in our study, no adverse events were reported despite administering the highest dose of 9 g/day. Consequently, SBT emerges as a potentially new alternative for XDR-AB infection and and further investigations into high-dose sulbactam single agent formulations are warranted.

However, there are still some limitations in our study. Firstly, this is a retrospective study, although we just included 52 patients, the sample size is already relatively large for sulbactam single agent formulation in treating XDR-AB infection. Secondly, this study includes patients with co-infections due to the limited number of patients with simple XDR-AB infection, which may have impacted the results.

## Conclusion

Sulbactam, especially the high dose of SBT maybe a new alternative to XDR-AB infection treatment, notably in intracranial infection. Longer treatment duration of SBT positively correlates with both survival and a favorable clinical response. Patients with a high CrCl might have an unfavorable clincal response.

## Electronic supplementary material

Below is the link to the electronic supplementary material.

Supplementary Material 1

## Data Availability

No datasets were generated or analysed during the current study.
