# Supplementary material for: A retrospective study of the efficacy of sulbactam in the treatment of patients with extensively drug-resistant Acinetobacter baumannii infections
Source: Infection. 2024 Jul 23;52(6):2445–54. doi: 10.1007/s15010-024-02307-9 (PMC11621167; doi:10.1007/s15010-024-02307-9)
Supplement: Supplementary file 1 — Supplementary Material 1 [file 15010_2024_2307_MOESM1_ESM.docx]

Supplementary Table 1 Baseline characteristics of patients with favorable and unfavorable clinical response

| Variables | Favorable clinical  response（n = 31） | Unfavorable clinical  response（n = 21） | *P*-value |
| --- | --- | --- | --- |
| Demographic parameters |  |  |  |
| Age, yr (mean ± SD) | 55.87 ± 19.61 | 60.81 ± 16.50 | 0.348 |
| Male gender (n, %) | 21 (67.7%) | 14 (66.7%) | 0.935 |
| BMI (mean ± SD) | 22.88 ± 3.40 | 25.21 ± 2.88 | 0.013* |
| Concomitant diseases (n, %) |  |  |  |
| Hepatic dysfunction | 7 (22.6 %) | 4 (19.0 %) | 1.000 |
| Renal insufficiency | 5 (16.1%) | 3 (14.3%) | 1.000 |
| Chronic respiratory disease (n, %) | 11 (35.5 %) | 12 (57.1 %) | 0.123 |
| Hypertension (n, %) | 11 (35.5 %) | 10 (47.6 %) | 0.382 |
| Other cardiovascular disease | 16 (51.6%) | 17 (81.0%) | 0.031* |
| Diabetes mellitus | 12 (38.7 %) | 5 (23.8 %) | 0.261 |
| Immune compromise | 5 (16.1%) | 8 (38.1%) | 0.073 |
| Infection site variables (n, %) |  |  |  |
| Pulmonary infection (n, %) | 23 (74.2 %) | 21 (100.0 %) | 0.032* |
| Plus bloodstream infection | 4 (12.9 %) | 7 (33.3 %) | 0.154 |
| Plus urinary tract infection | 0 (0.0 %) | 1 (4.8 %) | 0.843 |
| Bloodstream infection (n, %) | 6 (19.4 %) | 10 (47.6 %) | 0.030* |
| Intracranial infection (n, %) | 7 (22.6 %) | 2 (9.5 %) | 0.397 |
| Microbiology characteristics (n,%) |  |  |  |
| Coinfection variables |  |  |  |
| Klebsiella pneumoniae | 7 (22.6 %) | 5 (23.8 %) | 1.000 |
| Pseudomonas aeruginosa | 4 (12.9 %) | 3 (14.3 %) | 1.000 |
| Concomitant with GPB infection | 4 (12.9 %) | 9 (42.9 %) | 0.014* |
| Concomitant with fungi infection | 15 (48.4 %) | 12 (57.1 %) | 0.535 |
| Treatment |  |  |  |
| Length of hospitalization,  (days, median, IQR) | 48.0 (27.0 ~ 56.0) | 18.0 (12.5 ~ 41.1) | 0.012* |
| ICU duration (days, median, IQR) | 11.0 (0.0 ~31.0) | 17.0 (5.0 ~ 26.0) | 0.652 |
| Days of therapy  (days, median, IQR) | 11.0 (5.0 ~17.0) | 4.0 (2.5 ~ 8.0) | 0.002* |
| Hosptilized in ICU when SBT treated (n, %) | 15 (48.4 %) | 19 (90.5%) | 0.002* |
| APACHE II ≥ 15 score (n, %) | 15 (48.4 %) | 13 (61.9 %) | 0.337 |
| CrCl (ml/min, median, IQR) | 83.9 (39.3 ~ 163.8) | 80.6 (56.1 ~ 124.1) | 0.963 |
| Dual drug combination (n, %) | 24 (77.4 %) | 14 (66.7 %) | 0.391 |
| With Tigecycline | 12 (38.7 %) | 7 (33.3 %) | 0.693 |
| With polymyxin B | 5 (16.1 %) | 4 (19.0 %) | 1.000 |
| With carbapenems | 5 (16.1 %) | 2 (9.5 %) | 0.787 |
| With Minocycline | 1 (3.2 %) | 2 (9.5 %) | 0.727 |
| triple drug combination (n, %) | 7 (22.6 %) | 7 (33.3 %) | 0.391 |
| Dosage of sulbactam (g/day) | 4.0 (3.0 ~6.0) | 3.0 (3.0 ~5.0) | 0.051 |
| ≥ 6g (n, %) | 13 (41.9 %) | 5 (23.8 %) | 0.178 |
| ＜ 6g (n, %) | 18 (58.1%) | 16 (76.2 %) | 0.178 |

Note: *P < 0.05, there was statistically significant difference.

Abbreviations: SD, standard deviation; BMI, body mass index; IQR, interquartile range; GPB, gram-positive bacterium; APACHE, acute physiology and chronic health evaluation; CrCl, creatinine clearance; ICU, intensive care unit.

Supplementary Table 2 Univariate analysis of factors associated with all-cause mortality by day 28 and clinical response by day 14

| Variables | 28-day mortality | | 14-day clinical response | |
| --- | --- | --- | --- | --- |
|  | *P*-value | OR-value | *P*-value | OR-value |
| Age | 0.033* | 1.040 (1.003 - 1.079) | 0.341 | 0.985 (0.955 – 1.016) |
| BMI | 0.263 | 1.017 (0.927 - 1.321) | 0.020* | 0.786 (0.642 – 0.963) |
| Underlying respiratory disease | 0.041* | 3.429 (1.053 – 11.159) | 0.126 | 0.413 (0.133 – 1.283) |
| Underlying other cardiovascular disease besides hypertension | - | - | 0.037* | 0.251 (0.069 – 0.918) |
| Underlying immune compromise | - | - | 0.080 | 0.313 (0.085 – 1.147) |
| Concomitant pulmonary infection | 0.998 | 1.228E+9 | - | - |
| Coinfected pulmonary and bloodstream infection | 0.044* | 4.229 (1.042 – 17.161) | 0.109 | 0.320 (0.080 – 1.287) |
| Concomitant bloodstream infection | 0.054 | 3.343 (0.979 – 11.412) | 0.035* | 0.264 (0.077 – 0.908) |
| Concomitant intracranial infection | 0.113 | 0.174 (0.020 – 1.513) | 0.235 | 2.771 (0.515– 14.908) |
| Concomitant GPB infection | 0.037* | 4.073 (1.091 – 15.203) | 0.019* | 0.198 (0.051 – 0.770) |
| Length of hospitalization | 0.001* | 0.923 (0.880 – 0.968) | 0.027* | 1.030 (1.004– 1.058) |
| Days of therapy | 0.010* | 0.825 (0.713 – 0.955) | 0.009* | 1.198 (1.047 – 1.371) |
| Hosptilized in ICU when treated in SBT | 0.038* | 4.444 (1.084 – 18.217) | 0.059 | 0.286 (0.078 – 1.047) |
| APACHE II ≥ 15 scores | 0.034* | 3.800 (1.108 – 13.034) | 0.339 | 0.577 (0.187– 1.782) |
| Dosage of SBT ≥ 6 g/day | 0.727 | 0.808 (0.243 – 2.680) | 0.183 | 2.311 (0.674 – 7.921) |
| Dosage of SBT | - | - | 0.070 | 1.331 (0.977 – 1.814) |
| CrCl | 0.191 | 0.994 (0.985 – 1.003) | 0.658 | 1.002 (0.995 – 1.009) |

Note: *P < 0.05, there was statistically significant difference.

Abbreviations: SD, standard deviation; BMI, body mass index; GPB, gram-positive bacterium; APACHE, acute physiology and chronic health evaluation; CrCl, creatinine clearance; ICU, intensive care unit.

Supplementary Table 3 Baseline characteristics of patients on high dose and normal dose

| Variables | Dosage ≥ 6g  （n = 18） | Dosage ＜ 6g  （n = 34） | *P*-value |
| --- | --- | --- | --- |
| Demographic parameters |  |  |  |
| Age, yr (mean ± SD) | 55.78 ± 17.64 | 58.97 ± 18.97 |  |
| Male gender (n, %) | 12 (66.7 %) | 23 (67.6 %) | 0.943 |
| BMI (mean ± SD) | 24.29 (21.9 ~ 26.3) | 24.49 (20.1 ~ 25.8) |  |
| Concomitant diseases (n, %) |  |  |  |
| Hepatic dysfunction | 3 (16.7 %) | 8 (23.5 %) | 0.826 |
| Renal insufficiency | 5 (27.8 %) | 3 (8.8 %) | 0.162 |
| Chronic respiratory disease (n, %) | 7 (38.9 %) | 16 (47.1 %) | 0.573 |
| Hypertension (n, %) | 5 (27.8 %) | 16 (47.1 %) | 0.178 |
| Other cardiovascular disease | 10 (55.6 %) | 23 (67.6 %) | 0.389 |
| Diabetes mellitus | 8 (44.4 %) | 9 (26.5 %) | 0.189 |
| Immune compromise | 3 (16.7 %) | 10 (29.4%) | 0.501 |
| Infection site variables (n, %) |  |  |  |
| Pulmonary infection (n, %) | 15 (83.3 %) | 29 (85.3 %) | 1.000 |
| Bloodstream infection (n, %) | 7 (38.9 %) | 9 (26.5 %) | 0.356 |
| Intracranial infection (n, %) | 4 (22.2 %) | 5 (14.7 %) | 0.767 |
| Microbiology characteristics (n,%) |  |  |  |
| Coinfection variables |  |  |  |
| Klebsiella pneumoniae | 6 (33.3 %) | 6 (17.6 %) | 0.352 |
| Pseudomonas aeruginosa | 4 (22.2 %) | 3 (8.8 %) | 0.358 |
| Concomitant with GPB infection | 4 (22.2 %) | 9 (26.5 %) | 1.000 |
| Concomitant with fungi infection | 9 (50.0 %) | 18 (52.9 %) | 0.840 |
| Treatment |  |  |  |
| Length of hospitalization,  (days, median, IQR) | 31.0 (16.0 ~ 52.0) | 35.0 (17.0 ~ 59.3) | 1.000 |
| ICU duration (days, median, IQR) | 13.0 (1.5 ~31.3) | 14.0 (0.0 ~ 25.3) | 0.742 |
| Days of therapy  (days, median, IQR) | 7.5 (2.8 ~14.5) | 6.0 (3.0 ~ 13.3) | 0.915 |
| Hosptilized in ICU when SBT treated (n, %) | 10 (55.6 %) | 24 (70.6 %) | 0.278 |
| APACHE II ≥ 15 score (n, %) | 9 (50.0 %) | 19 (55.9 %) | 0.686 |
| CrCl < 30 ml/min | 0 (0.0 %) | 4 (11.8 %) | 0.333 |
| Dual drug combination (n, %) | 12 (66.7 %) | 26 (76.5 %) | 0.667 |
| With Tigecycline | 4 (22.2 %) | 15 (44.1 %) | 0.119 |
| With polymyxin B | 5 (27.8 %) | 4 (11.8 %) | 0.286 |
| With carbapenems | 2 (11.1 %) | 5 (14.7 %) | 1.000 |
| With Minocycline | 1 (5.6 %) | 2 (5.9 %) | 1.000 |
| triple drug combination (n, %) | 6 (33.3 %) | 8 (23.5 %) | 0.667 |
| Clinical outcome |  |  |  |
| 28-day survival | 12 (66.7 %) | 21 (61.8 %) | 0.727 |
| 14-day clinical response | 13 (72.2 %) | 18 (52.9 %) | 0.178 |
| Time to response ^a^  (days, median, IQR) | 5.0 (2.5 ~ 8.0) | 8.5 (4.8 ~ 12.0) | 0.042* |

a. The statistic of time to response had excluded the patients with 14-day unfavorable response (n = 31).

Abbreviations: SD, standard deviation; BMI, body mass index; IQR, interquartile range; GPB, gram-positive bacterium; APACHE, acute physiology and chronic health evaluation; CrCl, creatinine clearance; ICU, intensive care unit.
